# Supplementary material for: Single-cell analysis reveals dynamics of human B cell differentiation and identifies novel B and antibody-secreting cell intermediates
Source: eLife. 2023 Mar 2;12:e83578. doi: 10.7554/eLife.83578 (PMC10005767; doi:10.7554/eLife.83578)
Supplement: Supplementary file 1. [file elife-83578-supp1.docx]

**Supplementary file 1.** Primer sequences

| Genes |  | Sequence (5’-3’) |
| --- | --- | --- |
| *18S-rRNA* | Forward  Reverse | CGGCTACCACATCCAAGGAA  GCTGGAATTACCGCGGCT |
| *PRDM1* | Forward  Reverse | AACGTGTGGGTACGACCTTG  ATTTTCATGGTCCCCTTGGT |
| *XBP1* | Forward  Reverse | CCGCAGCACTCAGACTACG  TGCCCAACAGGATATCAGACT |
| *IRF4* | Forward  Reverse | CCACCACTGGCAAGGCCCAG  GCAGCCGGCAGTCTGAGAACG |
| *PAX5* | Forward  Reverse | ACGCTGACAGGGATGGTG  CCTCCAGGAGTCGTTGTACG |
| *BACH2* | Forward  Reverse | TTGCCTGAGGAGGTCACAG  ACAGGCCATCCTCACTGTTC |
